# Laboratory colonization of the European invasive mosquito *Aedes* (*Finlaya*) koreicus

**DOI:** 10.1186/s13071-017-2010-2

**Published:** 2017-02-10

**Authors:** Silvia Ciocchetta, Jonathan M. Darbro, Francesca D. Frentiu, Fabrizio Montarsi, Gioia Capelli, John G. Aaskov, Gregor J. Devine

**Affiliations:** 1 QIMR Berghofer Medical Research Institute, Royal Brisbane Hospital, Brisbane, Australia; 2 Queensland University of Technology, Brisbane, Australia; 3 Istituto Zooprofilattico Sperimentale delle Venezie, Padova, Italy

**Keywords:** *Aedes koreicus*, Invasive mosquito species, Laboratory colonization, Fecundity index, Pupae differentiation

## Abstract

**Background:**

*Aedes* (*Finlaya*) *koreicus* (Edwards) is a mosquito that has recently entered Europe from Asia. This species is considered a potential threat to newly colonized territories, but little is known about its capacity to transmit pathogens or ability to compete with native mosquito species. The establishment of a laboratory colony is a necessary first step for further laboratory studies on the biology, ecology and vector competence of *Ae. koreicus*.

**Results:**

A self-mating colony was established at QIMR Berghofer Medical Research Institute (Brisbane, Australia) from eggs of the F1 progeny of individuals collected as free-living larvae in northeastern Italy (Belluno province). Mosquitoes are currently maintained on both defibrinated sheep blood provided via an artificial membrane system and human blood from volunteers. Larvae are maintained in rain water and fed with Tetramin^®^ fish food (©2015 Spectrum Brands - Pet, Home and Garden Division, Tetra-Fish). Morphometric measurements related to body size were taken and a fecundity index, based on wing length, was calculated. An in vivo technique for differentiating male and female pupae has been optimized. Our findings provide the basis for further studies on the ecology and physiology of *Ae. koreicus*.

**Conclusion:**

We describe the establishment of an *Ae. koreicus* colony in the laboratory and identify critical requirements for the maintenance of this mosquito species under artificial conditions. The laboratory colony will facilitate studies investigating the vector potential of this species for human pathogens.

## Introduction

*Aedes* (*Finlaya*) *koreicus* (Edwards, 1917) is a newly-invading mosquito species [1] from South-east Asia [2] that has been repeatedly detected in Europe in recent years: Belgium in 2008 [3], Italy in 2011 [4, 5], Russia [6] and Switzerland in 2013 [7] and Germany in 2015 [8]. These new country records are reported in addition to the documented and rapid spread of *Ae. koreicus* in Italy over the last four years [9], indicating this species’ potential to spread and establish throughout Europe [10].

Despite the importance of some members of the genus *Aedes* as vectors of human viral pathogens [11], the behavior of *Ae. koreicus* and its status as a vector of arboviruses remains largely unknown [4, 12]. A recent study confirmed its potential to vector the parasitic nematode *Dirofilaria immitis* [13], but the risk of transmission of viruses such as dengue, chikungunya and Zika by *Ae. koreicus* is poorly understood. Its status as a potential vector is also complicated by some historical confusion regarding its native range. The species has often been confused with *Aedes japonicus japonicus* (Reinert, 2000) [4, 14].

Although colonies of others *Aedes* species have been established in the laboratory in the past [15–18], no successful self-mating *Ae. koreicus* colonies have been reported in the literature and thus no satisfactory method of rearing this species has been yet described. In this study, several experiments have been conducted to describe the successful rearing conditions of our colony and the key biological attributes of our laboratory-reared *Ae. koreicus* (e.g. development times, fecundity and egg hatching rates).

As part of our investigation, the technique described by Moorefield [19] was adapted to successfully differentiate *Ae. koreicus* male and female at the pupal stage with the goal of creating cohorts of virgin mosquitoes. The capacity to obtain virgin cohorts is of considerable utility when designing experiments that investigate competitive behaviour and mating interference between invasive and native mosquito species such as satyrisation [20–23].

Wing length is often an accurate indicator of fecundity in mosquitoes [24–28] and numerous studies have exploited this relationship to investigate mosquito ecology and behavior [29–32]. To facilitate similar studies on *Ae. koreicus* we describe the fecundity-size relationship of our newly established colony.

The methodologies described in this report can now be used to establish further colonies and facilitate studies on vector competence and inter-specific competition with native species and other invasive species.

## Methods

### Effect of temperature on *Ae. koreicus* egg hatching and development

In a first attempt to colonize *Ae. koreicus* in laboratory at Istituto Zooprofilattico Sperimentale delle Venezie (IZSVe), Italy, mosquitoes were reared following the protocol of Williges et al. [33] due to the phylogenetic proximity of this species to *Ae. japonicus* [12, 34]. The rearing conditions were as follows: 26 ± 1 °C temperature, 65 ± 5% relative humidity and a 16-h light: 8-h dark photocycle, without crepuscular periods.

Due to considerable rearing problems under these conditions (a lack of oviposition and colony decline), we hypothesized that temperature was affecting our colonization success. *Aedes koreicus* egg development was compared, from hatching to adult stage, at two different rearing temperatures (23 ± 1 °C and 26 ± 1 °C). The temperature choice of 23 ± 1 °C was based on the average summer temperature of the native range of *Ae. koreicus* in South Korea and the average temperature in which the species is currently present in the mountainous area of Belluno, Italy [5].

Eggs were collected from field (IZS Belluno) (46.1477339°N, 12.2046886°E) using Masonite^®^ sticks (as oviposition substrates) placed partially submerged in rainwater on the edges of 60 l black bins (ABM Italia S. p. A.). Once collected, the eggs were hatched in the laboratory over 17 days (8–25 July 2014) in rainwater. During hatching, 204 eggs hatched were held at the higher temperature range while 233 eggs were exposed to the lower temperature range. Larvae were fed on an aqueous solution of ground Tetramin^®^ fish food (0.125 g/ml) *ad libitum* (dry Tetramin^®^ fish food powder directly added to the trays was observed to cause excessive bacterial scum and larval death). Based on the results of this experiment, we continued to rear our *Ae. koreicus* colony at 23 ± 1 °C.

### Establishment of *Ae. koreicus* colony

Eggs from the initial Italian colony, reared in laboratory at IZSVe, were used to establish a new colony of *Ae. koreicus* at QIMR Berghofer Medical Research Institute under import permit IP 14001574. Rearing conditions for the new colony of *Ae. koreicus* were: 23 ± 1 °C temperature, 75 ± 5% relative humidity and a 12-h light: 12-h dark cycle, with crepuscular periods. Larvae were reared in 45 × 40 × 5 cm white plastic trays that contained approximately 5 l of rain water or de-chlorinated tap water never exceeding a density of 500 larvae per tray. They were fed on an aqueous solution of ground Tetramin^®^ fish food (0.125 g/ml) added to the trays, never exceeding the following amounts: 0.5 ml of Tetramin^®^ fish food solution for first and second instar larvae, 1 to 2 ml for third-instar larvae, and 2 ml for fourth-instar larvae.

In the first stages of larval development (L1 and L2), the aqueous food solution was provided every two days. Food was supplied daily during the subsequent development stages (L3 and L4). Water levels were maintained by adding fresh rain or de-chlorinated tap water to the trays. Pupae were individually ‘picked’ from larval trays using a 1.5 ml pipette and transferred to the egg collection trays (© 2014 Genfac Plastics Pty Ltd, 18.3 × 15.2 × 6.5 cm). These trays contained rain water and Masonite^®^ sticks as oviposition substrates. Pupae density did not exceed 250 pupae per tray. Trays were placed inside adult colony cages (BugDorm^®^ Insect Rearing Cage, 30 × 30 × 30 cm) in preparation for adult emergence, mating and oviposition. Once emerged, adult mosquitoes were provided with 10% sucrose solution *ad libitum* and allowed to feed on the arms of a human volunteer (with QIMR Berghofer IRB approval) or defibrinated sheep blood (Thermo Fisher Scientific^®^ Aust Pty Ltd) supplied through glass membrane feeders covered by a porcine intestinal membrane [35]. No forced mating was required. Masonite^®^ sticks with *Ae. koreicus* eggs were routinely placed for no longer than 10 min on dry paper towels to absorb excess water. They were then stored in anti-leak plastic bags that were sealed to prevent desiccation as suggested by Crampton et al. [36] and maintained at 23 ± 1 °C.

### *Aedes koreicus* egg storage and embryo development

To determine if the low hatching rate observed in our colony was due to poor storage conditions, we investigated embryo development of our stored eggs. After 14 days of storage in a sealed anti-leak plastic bag, one Masonite^®^ stick holding 1,189 eggs was observed under the stereoscope to assess damage or contamination. Following the evaluation of egg integrity under the stereoscope, a segment of the Masonite^®^ stick holding a total of 95 undamaged eggs was then bleached for 30 min in a 50% bleach solution modifying the method used by Trpiš [37], and observed under the stereoscope to confirm embryogenesis. The remaining 1,094 eggs were submerged in a hatching tray with 5 l of rain water. Larvae were fed with the typical colony rearing food regimen and the number of adults obtained was recorded.

### Sexual dimorphism in *Ae. koreicus* pupae

Morphological features of the genital lobe of *Ae. koreicus* pupae were investigated as a means of distinguishing males from females. Pupae were inspected in a water droplet under 20× magnifications. Coverslips were not used as they damaged the pupae.

### *Aedes koreicus* fecundity-size relationship evaluation

To determine whether wing length could be used as an indicator of fecundity in *Ae. koreicus,* larvae were divided between four trays of 100 larvae each, three days after hatching. The volume of water per tray was 5 l. To create mosquito cohorts of different sizes, we applied different feeding regimes. One group was provided with aqueous solution of ground Tetramin^®^ fish food (0.125 g/ml) *ad libitum*, larvae from three other groups were fed with the same solution at various ratios: first instars larvae were given respectively 0.05, 0.1 and 0.2 ml fish food solution per tray; second instars larvae were fed 0.1, 0.2, and 0.4 ml of food per tray; third instars larvae were fed 0.15, 0.3, and 0.6 ml of food per tray and fourth instars larvae were fed 0.2, 0.4, and 0.8 ml of food per tray daily.

Adults that emerged from these rearing trays were blood-fed to repletion on a human host approximately 5 days after eclosion. Five days after blood-feeding, female mosquitoes were removed from the cage and killed (using carbon dioxide). As a proxy of body size, the length from the arculus to the wing tip, excluding the fringe scales, was measured. Both wings were removed and dry mounted on a glass microscope slide. In cases where the right and left wings differed in size, a mean length was calculated [24–28].

Ovaries were dissected in a drop of phosphate-buffered saline (PBS) on a glass microscope slide under a stereoscope at a magnification of 10×. The number of mature follicles (stage IVb and V) were counted [24, 26]. Ovary development stages were classified according to Clements & Boocock [38] modified from Christophers [39].

### Data analysis

*Aedes koreicus* eggs development at two different rearing temperatures (23 ± 1 °C and 26 ± 1 °C) was compared using the *X* ^2^ test (Prism GraphPad 6^®^). To determine the fecundity-size correlation a linear regression analysis (Prism GraphPad 6^®^) was performed using the number of mature follicles and wing length.

## Results and discussion

### Effect of temperature on *Ae. koreicus* egg hatching and development

From a total of 233 eggs reared at IZSVe laboratory in Italy at 23 ± 1 °C, 39.41% reached the adult stage (*n* = 93; 37 males, 56 females). By contrast, the percentage of adults obtained from the 204 eggs reared at the same Institute at 26 ± 1 °C was just 3.43% (*n* = 7; 3 males, 4 females). Significantly more *Ae. koreicus* adults developed at the lower temperature than at the higher temperature (*χ* ^2^ = 82.04, *P* < 0.0001). These egg cohorts developed slowly with a great deal of variation in emergence times (over a period of 17 days). Following this initial finding, the rearing temperature of the *Ae. koreicus* colony in Italy was adjusted to 23 ± 1 °C, and after 3 months 8,860 eggs had been collected. These were sent to QIMR Berghofer Medical Research Institute to start a new *Ae. koreicus* colony for vector competence studies.

### Establishment of *Ae. koreicus* colony

**Fig. 1:**
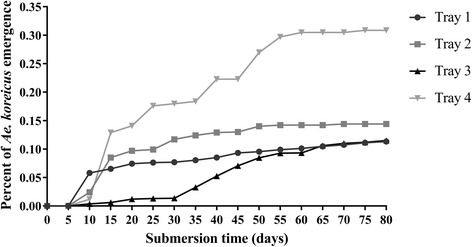
*Aedes koreicus* pupae development measured over 80 days of submersion in four different trays

Development times and hatching rates of the QIMR Berghofer Medical Research Institute colony at 23 ± 1 °C are reported in Table 1. This species shows a low percentage of pupae obtained nine days after submersion of eggs. The eggs can remain submerged but viable for very long periods. The cumulative proportion of pupae obtained from submerged eggs over a period of 80 days is shown in Fig. 1. Long viability of submerged eggs could be due to embryo dormancy, a demonstrated survival strategy in other mosquitoes [40]. The emergence of adults over a long period after water submersion could represent a mechanism that permits coexistence with competing species. Another potential competitive advantage is earlier hatching during the spring season, observed when *Ae. koreicus* shares the same breeding sites with *Ae. albopictus* [5].

**Table 1:** Development parameters for *Ae. koreicus* reared at a temperature of 23 ± 1 °C

Time to pupation (days ± SE)	Time to pupae eclosion (days ± SE)	Interval between blood meal and oviposition (days ± SE)	Hatching percentage (% ± SE)
9.29 ± 0.18	3.43 ± 0.3	11.5 ± 3.5	10.39 ± 2.09

*Abbreviation*: *SE* standard error of the mean

### *Aedes koreicus* egg storage and embryo development

**Fig. 2:**
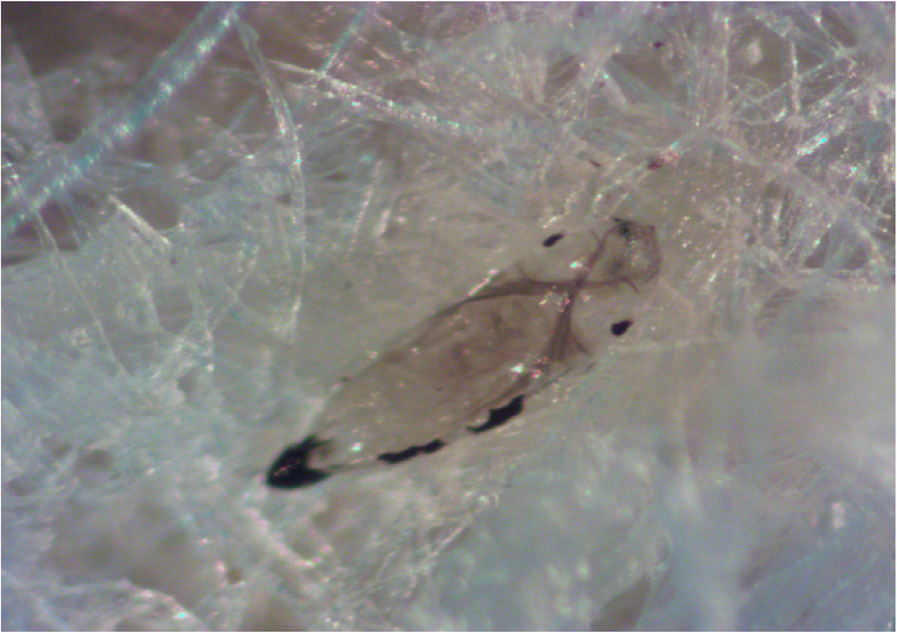
*Aedes koreicus* fully formed embryo identifiable after egg shell clearing process

From the observation of 1,189 eggs at 14 days post storage, a total of 5 eggs were found to be desiccated and 3 eggs were hatched, with all the other eggs appearing normal. After bleaching a portion of the eggs (*n* = 95) to determine the embryo development status, it was possible to observe a total of 83 mature embryos under the stereoscope (87.37%, *n* = 95). Each embryo was considered mature when eye-spots and thoracic and abdominal hair tufts were clearly visible. This is typical of a fully developed embryo [41] as shown in Fig. 2. During the bleaching process 12 eggs were lost in the media and were not evaluated. Although embryonation was confirmed, only 25 first-instar larvae were observed after 36 h of water submersion of the remaining eggs (2.28%, *n* = 1,094). Pupation was observed from day 9 onwards. After 14 days, of the 25 original larvae, 20 had reached adult stage. This study confirmed that our low hatching rate is not due to inadequate storage of eggs.

### Sexual dimorphism in *Ae. koreicus* pupae

**Fig. 3:**
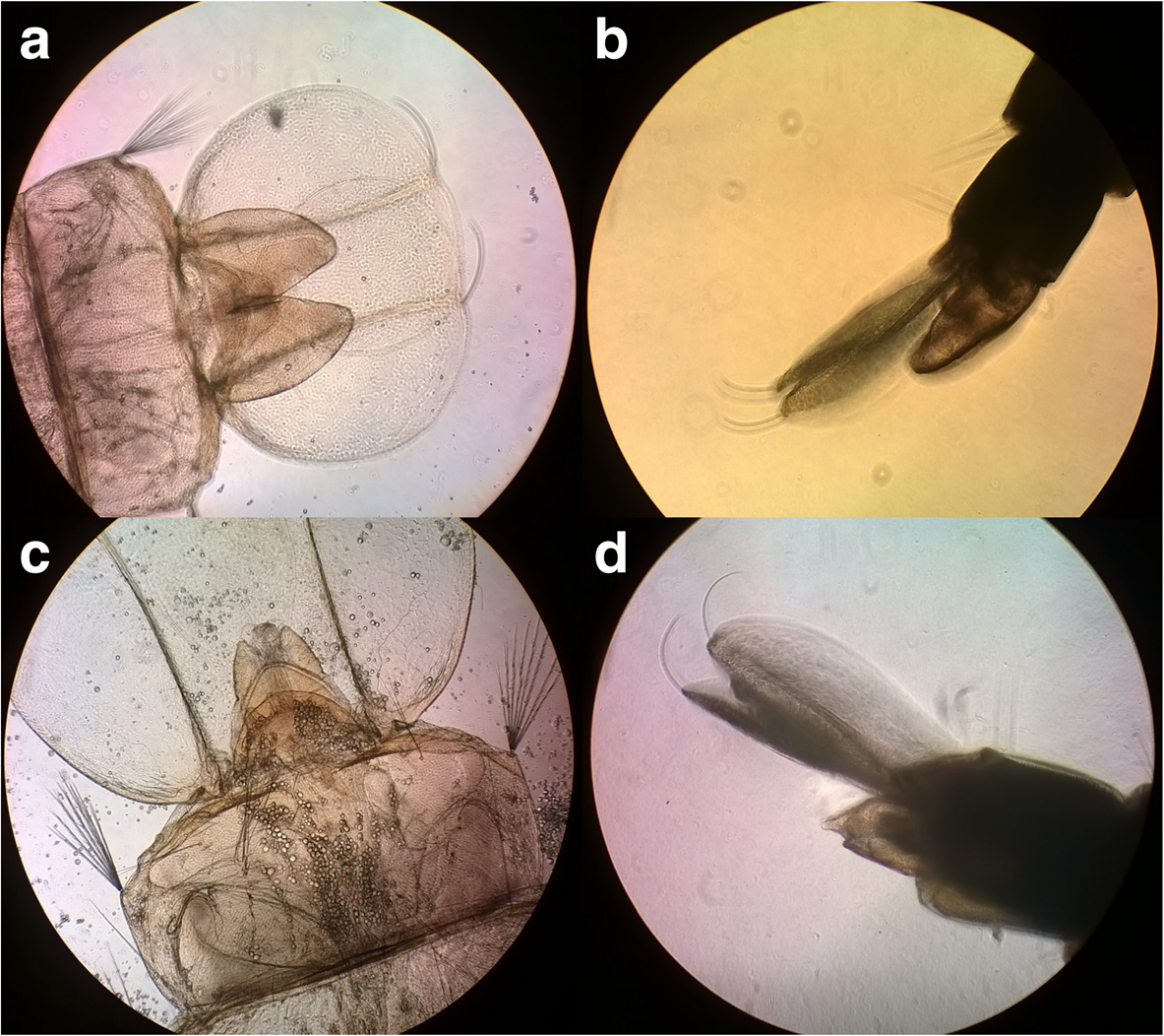
*Aedes koreicus* male and female genital lobe. **a** Male genital lobe after dissection and after observation of pupae alive in a water drop as per technique described in the text (**b**). **c** Female genital lobe after dissection and after observation of pupae alive in a water drop as per technique described in the text (**d**)

The characteristic conformation of the genital lobe after dissection and in live pupae allows distinguishing sex as shown in Fig. 3. Following examination, pupae were individually placed in water containers and emerging adults sexed. Observation of the genital lobe in pupae allowed 100% successful separation of male and female *Ae. koreicus*.

### *Aedes koreicus* fecundity-size relationship evaluation

**Fig. 4:**
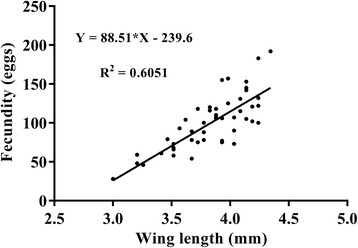
Relationship between wing length and fecundity for *Aedes koreicus*

A strong relationship was detected between fecundity (number of eggs) and female size (with wing length as a proxy) (Fig. 4; *R* ^2^ = 0.6051, *P* < 0.0001; *n* = 51). This indicates that the size of individuals collected from the field can be related to fecundity.

## Conclusions

We provide the first report of laboratory colonization of *Ae. koreicus*. The information provided promotes a better understanding of the biology of laboratory colonies of this European invader. It will help facilitate investigations on ecology, competition and vectorial capacity in the laboratory. This will help inform potential public health or ecological issues associated with its continuing spread.

## References

[1] Schaffner F, Bellini R, Petrić D, Scholte E-J, Zeller H, Rakotoarivony LM (2013). Development of guidelines for the surveillance of invasive mosquitoes in Europe. Parasit Vectors.

[2] Tanaka K, Saugstad ES, Mizusawa K (1979). A revision of the adult and larval mosquitoes of Japan (including the Ryukyu Archipelago and the Ogasawara Islands) and Korea (Diptera: Culicidae). Contrib Am Entomol Inst (Ann Arbor).

[3] Versteirt V, De Clercq E, Fonseca D, Pecor J, Schaffner F, Coosemans M (2012). Bionomics of the established exotic mosquito species *Aedes koreicus* in Belgium, Europe. J Med Entomol.

[4] Capelli G, Mathis A, Di Luca M, Romi R, Russo F, Drago A (2011). First report in Italy of the exotic mosquito species *Aedes* (*Finlaya*) *koreicus*, a potential vector of arboviruses and filariae. Parasit Vectors.

[5] Montarsi F, Ravagnan S, Ciocchetta S, Russo F, Capelli G, Martini S (2013). Distribution and habitat characterization of the recently introduced invasive mosquito *Aedes koreicus* (*Hulecoeteomyia koreica*), a new potential vector and pest in north-eastern Italy. Parasit Vectors.

[6] Bezzhonova OV, Patraman IV, Ganushkina LA, Vyshemirskii OI, Sergiev VP (2014). The first finding of invasive species *Aedes* (*Finlaya*) *koreicus* (Edwards, 1917) in European Russia. Med Parazitol (Mosk).

[7] Suter T, Flacio E, Fariña BF, Engeler L, Tonolla M, Müller P (2015). First report of the invasive mosquito species *Aedes koreicus* in the Swiss-Italian border region. Parasit Vectors.

[8] Werner D, Zielke D, Kampen H. First record of *Aedes koreicus* (Diptera: Culicidae) in Germany. Parasitol Res. 2016;115(3):1331–1334. 10.1007/s00436-015-4848-6 26614356

[9] Montarsi F, Drago A, Martini S, Calzolari M, De Filippo F, Bianchi A (2015). Current distribution of the invasive mosquito species, *Aedes koreicus* (*Hulecoeteomyia koreica*) in northern Italy. Parasit Vectors.

[10] Marcantonio M, Metz M, Baldacchino F, Arnoldi D, Montarsi F, Capelli G (2016). First assessment of potential distribution and dispersal capacity of the emerging invasive mosquito *Aedes koreicus* in Northeast Italy. Parasit Vectors.

[11] Schaffner F, Medlock JM, Van Bortel W (2013). Public health significance of invasive mosquitoes in Europe. Clin Microbiol Infect.

[12] Cameron EC, Wilkerson RC, Mogi M, Miyagi I, Toma T, Kim HC (2010). Molecular phylogenetics of *Aedes japonicus*, a disease vector that recently invaded Western Europe, North America, and the Hawaiian islands. J Med Entomol.

[13] Montarsi F, Ciocchetta S, Devine G, Ravagnan S, Mutinelli F, di Regalbono AF (2015). Development of *Dirofilaria immitis* within the mosquito *Aedes* (*Finlaya*) *koreicus*, a new invasive species for Europe. Parasit Vectors.

[14] Versteirt V, Pecor JE, Fonseca DM, Coosemans M, Van Bortel W (2012). Confirmation of *Aedes koreicus* (Diptera: Culicidae) in Belgium and description of morphological differences between Korean and Belgian specimens validated by molecular identification. Zootaxa.

[15] Fay RW (1964). The biology and bionomics of *Aedes aegypti* in the laboratory. Mosq News.

[16] Hartberg W, Gerberg E (1971). Laboratory colonization of *Aedes simpsoni* (Theobald) and *Eretmapodites quinquevittatus* Theobald. Bull World Health Organ.

[17] Deshmukh P, Guttikar S, Guru P, Bhat U (1973). Colonization of two species of mosquitoes, *Aedes novalbopictus* and *Aedes w-albus*. Indian J Med Res.

[18] Choochote W (1987). A note on laboratory colonization of *Aedes* (*Muscidus*) *quasiferinus* Mattingly 1961, Amphur Muang Chiang Mai, Northern Thailand. 1987. Southeast Asian J Trop Med Public Health.

[19] Moorefield H (1951). Sexual dimorphism in mosquito pupae. Mosq News.

[20] Bargielowski I, Lounibos L (2014). Rapid evolution of reduced receptivity to interspecific mating in the dengue vector *Aedes aegypti* in response to satyrization by invasive *Aedes albopictus*. Evol Ecol.

[21] Bargielowski IE, Lounibos LP, Carrasquilla MC (2013). Evolution of resistance to satyrization through reproductive character displacement in populations of invasive dengue vectors. Proc Natl Acad Sci USA.

[22] Carrasquilla MC, Lounibos LP (2015). Satyrization without evidence of successful insemination from interspecific mating between invasive mosquitoes. Biol Lett.

[23] Tripet F, Lounibos LP, Robbins D, Moran J, Nishimura N, Blosser EM (2011). Competitive reduction by satyrization? Evidence for interspecific mating in nature and asymmetric reproductive competition between invasive mosquito vectors. Am J Trop Med Hyg.

[24] Hugo L, Kay B, Ryan P (2003). Autogeny in *Ochlerotatus vigilax* (Diptera: Culicidae) from Southeast Queensland, Australia. J Med Entomol.

[25] Blackmore MS, Lord CC (2000). The relationship between size and fecundity in *Aedes albopictus*. J Vect Ecol.

[26] Armbruster P, Hutchinson RA (2002). Pupal mass and wing length as indicators of fecundity in *Aedes albopictus* and *Aedes geniculatus* (Diptera: Culicidae). J Med Entomol.

[27] Lymo EO, Takken W (1993). Effects of adult body size on fecundity and the pre-gravid rate of *Anopheles gambiae* females in Tanzania. Med Vet Entomol.

[28] Lounibos L, Larson V, Morris C (1990). Parity, fecundity and body size of *Mansonia dyari* in Florida. J Am Mosq Control Assoc.

[29] Alto BW, Lounibos LP, Higgs S, Juliano SA (2005). Larval competition differentially affects arbovirus infection in *Aedes* mosquitoes. Ecology.

[30] Alto BW, Lounibos LP, Mores CN, Reiskind MH (2008). Larval competition alters susceptibility of adult *Aedes* mosquitoes to dengue infection. Proc Biol Sci.

[31] Reiskind M, Lounibos L (2009). Effects of intraspecific larval competition on adult longevity in the mosquitoes *Aedes aegypti* and *Aedes albopictus*. Med Vet Entomol.

[32] Kesavaraju B, Leisnham PT, Keane S, Delisi N, Pozatti R (2014). Interspecific competition between *Aedes albopictus* and *A. sierrensis*: potential for competitive displacement in the Western United States. PLoS One.

[33] Williges E, Farajollahi A, Scott JJ, Mccuiston LJ, Crans WJ, Gaugler R (2008). Laboratory colonization of *Aedes japonicus japonicus*. J Am Mosq Control Assoc.

[34] Versteirt V, Nagy Z, Roelants P, Denis L, Breman F, Damiens D (2015). Identification of Belgian mosquito species (Diptera: Culicidae) by DNA barcoding. Mol Ecol Res.

[35] Rutledge L, Ward R, Gould D (1964). Studies on the feeding response of mosquitoes to nutritive solutions in a new membrane feeder. Mosq News.

[36] Crampton J, Beard C, Louis C (1997). The molecular biology of insect disease vectors: a methods manual.

[37] Trpiš M (1970). A new bleaching and decalcifying method for general use in zoology. Can J Zool.

[38] Clements A, Boocock M (1984). Ovarian development in mosquitoes: stages of growth and arrest, and follicular resorption. Physiol Entomol.

[39] Christophers SR (1911). The development of the egg follicle in anophelines. Paludism.

[40] Minakawa N, Githure JI, Beier JC, Yan G (2001). Anopheline mosquito survival strategies during the dry period in western Kenya. J Med Entomol.

[41] Decoursey JD, Webster A (1952). A method of clearing the chorion of *Aedes sollicitans* (Walker) eggs and preliminary observations on their embryonic development. Ann Entomol Soc Am.

